# Evaluating the Stress-Strain Relationship of the Additively Manufactured Lattice Structures

**DOI:** 10.3390/mi14010075

**Published:** 2022-12-27

**Authors:** Long Zhang, Farzana Bibi, Imtiyaz Hussain, Muhammad Sultan, Adeel Arshad, Saqib Hasnain, Ibrahim M. Alarifi, Mohammed A. Alamir, Uzair Sajjad

**Affiliations:** 1 School of Metallurgy, Lanzhou Resources & Environment Voc-Tech University, Lanzhou 730123, China; 2 Department of Energy and Refrigerating Air-Conditioning Engineering, National Taipei University of Technology, Taipei 10608, Taiwan; 3 Department of Agriculture Engineering, Faculty of Agriculture Sciences & Technology, Bahauddin Zakariya University, Multan 60800, Pakistan; 4 Department of Mechanical and Construction Engineering, Faculty of Engineering and Environment, Northumbria University, Newcastle upon Tyne NE1 8ST, UK; 5 School of Chemical Engineering, University of New South Wales, Sydney, NSW 2052, Australia; 6 Department of Mechanical and Industrial Engineering, College of Engineering, Majmaah University, Al-Majmaah, Riyadh 11952, Saudi Arabia; 7 Department of Mechanical Engineering, College of Engineering, Jazan University, Jazan 45142, Saudi Arabia

**Keywords:** additive manufacturing, lattice structure, stress-strain relationship, Bayesian optimization, deep learning, explainable artificial intelligence, mechanical properties

## Abstract

Extensive amount of research on additively manufactured (AM) lattice structures has been made to develop a generalized model that can interpret how strongly operational variables affect mechanical properties. However, the currently used techniques such as physics models and multi-physics simulations provide a specific interpretation of those qualities, and are not general enough to assess the mechanical properties of AM lattice structures of different topologies produced on different materials via several fabrication methods. To tackle this problem, this study develops an optimal deep learning (DL) model based on more than 4000 data points, which has been optimized by analyzing three different hyper-parameters optimization schemes including gradient boost regression trees (GBRT), gaussian process (GP), and random forest (RF) with different data distribution schemes such as normal distribution, nth root transformation, and robust scaler. With the robust scaler and *n*th root transformation, the accuracy of the model increases from R^2^ = 0.85 (for simple distribution) to R^2^ = 0.94 and R^2^ = 0.88, respectively. After feature engineering and data correlation, the stress, unit cell size, total height, width, and relative density are chosen to be the input parameters to model the strain. The optimal DL model is able to predict the strain of different topologies of lattices (such as circular, octagonal, Gyroid, truncated cube, Truncated cuboctahedron, Rhombic do-decahedron, and many others) with decent accuracy (R^2^ = 0.936, MAE = 0.05, and MSE = 0.025). The parametric sensitivity analysis and explainable artificial intelligence (by using DeepSHAP library) based insights confirm that stress is the most sensitive input to the strain followed by the relative density from the modeling perspective of the AM lattices. The findings of this study would be helpful for the industry and the researchers to design AM lattice structures of different topologies for various engineering applications.

## 1. Introduction

Due to its remarkable mechanical qualities and manufacturing capabilities (adaptability, flexibility, and adjustability), additive manufacturing (AM) has recently attracted the attention of academia and industry [1,2]. In AM, a structure can be constructed “layer by layer” into the desired shape. Complex items can now be manufactured using AM [3]. The production of identical-sized products in accordance with theoretical design, available manufacturing techniques, and commercial obstacles are just a few of the difficulties that must be overcome [4]. Cost competitiveness, commercial alloy availability, and control over the microstructure evaluation are some of these difficulties. Additionally, the choice of AM methods, materials, topologies, and other variables results in products of totally different attributes [5]. As known, the stress-strain behavior of various topologies of the lattice structures fabricated by different AM methods is innately different [4]. These influences of different topologies, materials, and fabrication methods can be assessed by evaluating the strain. The stress-strain curves play a crucial role in determining the mechanical characteristics of the AM components. In addition, with the knowledge of strain, other parameters including deflection, energy absorption, stiffness, and like can be assessed. Keeping this in view, it is challenging to evaluate the stress-strain relationship of different lattice structures fabricated by numerous AM methods. An overview of the experimental and simulation works to assess the stress-strain relationship of various topologies of AM lattice structures can be found in the literature (see Table 1).

The existing literature lacks such a general method to predict the stress-strain relationship for different topologies of lattice structures for different materials and AM methods. In line with this, a generalized methodology should be proposed that can tackle such a problem. Also, the proposed methodology should be equipped with the knowledge of parameter sensitivity analysis so that this knowledge can be used to assess more complex topologies created by more and more AM methods for numerous materials. As known, the mechanical properties of AM lattices are essentially determined by the qualities of the materials used to create them. Another consideration is the structure’s topology [6]. For a potential application, a material such as metals and alloys [7,8], composites, polymers, glass, ceramics, etc., and their lattice structures [8] are evaluated for their effectiveness in terms of their phase field performance [9], corrosion [10,11], mechanical properties, microstructure [12], fracture [13] as well as fatigue behavior [14] depending on that application. Such analyses necessitate the use of computational tools. A comprehensive review of modeling of AM lattices presents the advantages and shortcomings of the existing simulation methods to model the mechanical properties of AM lattice structures [15]. It has been reported that the existing approaches may not be effective for simulating mechanical performance in engineering applications due to the complicated geometry of AM lattice structures. In particular, for complex geometries and properties, this problem becomes more severe as the computational cost and accuracy of the simulation need to be improved significantly [15]. On the other hand, artificial intelligence, machine learning, and other computational techniques have proved their effectiveness for assessment, classification, optimization, and foreseeing time series data in various engineering applications [7,16,17]. More specifically, various AI and ML-based algorithms have been introduced recently to model the mechanical properties of additively manufactured honeycombs [18] and porous structures [19]. To the best of the authors’ knowledge, no such study has been published in the literature that uses an optimal deep learning framework based on the optimal set of hyper-parameters provided by Bayesian surrogate models and also assisted with the explainable artificial intelligence to evaluate the stress-strain relationship, perform sensitivity analysis, and interpret the predictions of the deep learning model by using explainable artificial intelligence (XAI) subject to numerous AM lattices created on different materials via different fabrication methods.

The goal of this work is to assess the stress-strain relationship of different topologies of AM lattice structures, perform the parameter sensitivity analysis, and highlight explainable artificial intelligence-based insights for modeling the strain of the different AM lattice structures. As a result, several AM lattices fabricated by different AM methods, materials, and topologies have been investigated in relation to various inputs such as unit cell size, stress, breadth, total height, relative density, and width. Initially, Bayesian surrogate models were created in order to produce a highly accurate deep learning model (based on fine-tuned hyper-parameters). The impact of each investigated input parameter on the mechanical parameter under consideration was then examined. Finally, the XAI was employed to generate interpretations for the DL predictions for each individual output and input parameter. The current study is an attempt to use artificial intelligence (AI) and explainable artificial intelligence (XAI) on AM lattice structures’ data for modeling the stress-strain relationship (for interpretations of the AI model’s predictions to better understand the trends and patterns as well as the quantitative and qualitative impacts of the input parameters in modeling the mechanical properties for the considered data range).

## 2. Materials and Methods

Initially, an experimental and simulation dataset of more than 4000 points has been collected from the literature (see Table 1). The collected data contains numerous topologies of lattice structures created on different materials such as metals and alloys, composites, ceramics, and polymers. The collected data clearly shows that different additive manufacturing methods including fused deposition modeling (FDM), multi-jet fusion (MJF), selective laser melting (SLM), stereolithography (SLA), direct metal laser sintering (DMLS), polyjet technology, and selective laser sintering (SLS) have been used to fabricate the considered lattice structures. Detailed references can be found in Table 1. The collected data is then used to develop a DL model.

**Table 1:** The investigated lattice structures, materials, and additive manufacturing methods.

Ref.	Lattice Structure	AM Method	Material	Schematic
[20]	Circular, octagonal, kelvin, rhombicuboctahedron, cubic	Multi Jet Fusion	Polymer	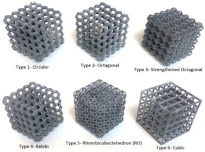
[21]	Gyroid	Selective Laser Melting	Metallic	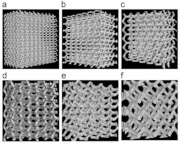
[22]	Cubic, Diamond, Truncated cube, Truncated cuboctahedron, Rhombic dodecahedron, Rhombicuboctahedron	SLM	Ti6Al4V-ELI powder (according to ASTM B348, grade 23) on top of a solid titanium substrate	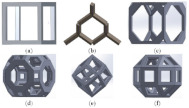
[23]	BCC-6H and BCC-12H	Laser Sintering	PA	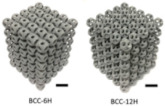
[24]	Local closed cell and global closed cell	Material extrusion process	PLA	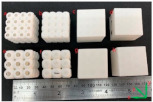
[25]	Kelvin lattice structures. 3 × 3 samples, 2 × 2 samples	Multi-jet fusion	Polyamide 12 (PA12)	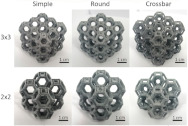
[25]	Octet truss lattice structures. 3 × 3 samples, 2 × 2 samples	Multi-jet fusion	Polyamide 12 (PA12)	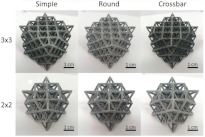
[26]	Face Centre Cube (FCC), Vertex Cube (VC), and Edge Centre Cube (ECC)	Selective laser melting		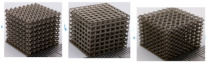
[27]	Pyramidal truss structure	Directed photo-curing technique	Thiol-ene polymer	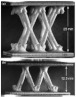
[28]	Diamond unit cell and periodic cellular lattice structures.	Direct metal laser sintering	AlSi10Mg	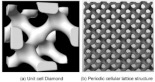
[29]	Diamond, Cubic, Body-centered, and Octet-truss	Stereo Lithography Apparatus (SLA)	Ceramic	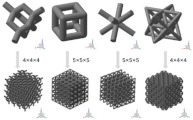
[30]	(BCC) body-centered cubic, (BCCZ) body-centered cubic with Z struts, (FCC) face-centered cubic, (FCCZ) face-centered cubic with Z struts, (FBCCZ) face and body-centered cubic with Z struts	Selective laser melting	AlSi12Mg	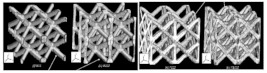
[31]	Polymeric lattice and Durazane-impregnated polymeric lattice, carbon lattice, and SiCN ceramic lattice	Stereo Lithography Apparatus (SLA)	Polymer-derived SiCN	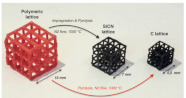
[32]	Triply periodic minimal surface (D and P surfaces)	Polyjet technology	Photopolymer ABS resin	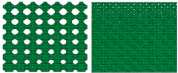
[33]	Cubic and diamond structures	SLM	Porous titanium (Ti6Al4V)	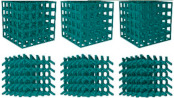
[34]	CLS struts	Fused deposition modeling	PLA	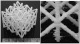

**Figure 1:**
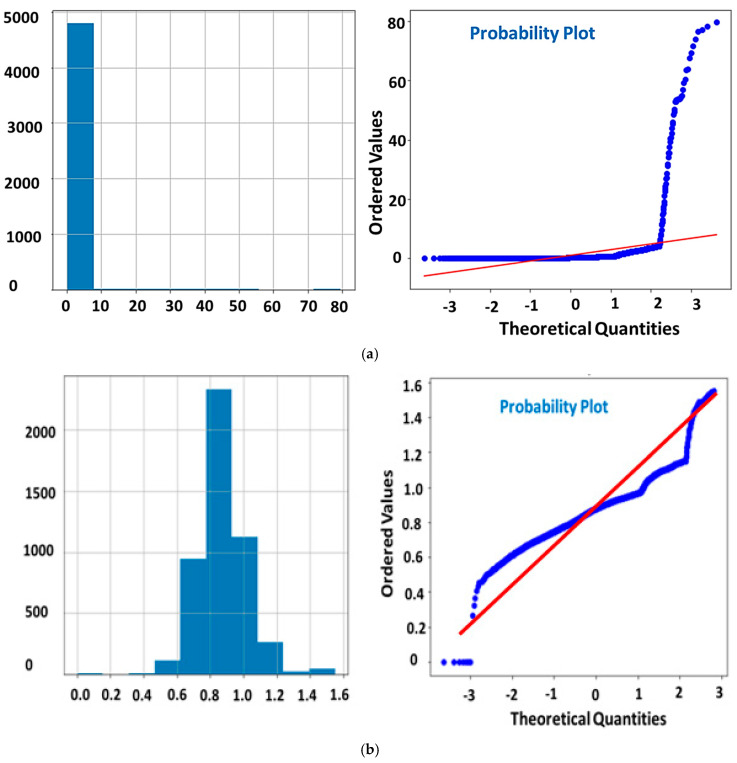
(**a**) Simple model data distribution and (**b**) data transformation with a robust scaler.

Three different Bayesian surrogate models including the gaussian process (GP), random forest (RF), and gradient boosting regression trees (GBRT) were employed to fine-tune the hyper-parameters. The details about these hyper-parameters tuning procedures can be found in prior works [35,36]. These surrogate models were evaluated for three different cases. The first case was named a simple model in which the actual data was used without any transformation. In the first case, these models (RF, GP, and GBRT) were used without any transformation. However, in the second and third cases, the original data was transformed by robust scaler and *n*th root transformation. The robust scaler transforms the data to Gaussian distribution. Data distribution in a simple model and data transformation with a robust scaler are presented in Figure 1. An overview of the employed methods to find the optimal deep learning model is given in Table 2, and their corresponding accuracies are depicted in terms of correlation coefficient (R^2^).

The given data is analyzed by quantile-quantile plots (Q-Q plots). It is a graphical plot used to compare two probability distributions. It is found that the data is negative or left skewed without transformation as shown in Figure 1a. If the original data without any transformation is used, the maximum correlation coefficient of 0.85 was found. However, when the data is transformed using a robust scaler, the negative skew distribution changed to no skew distribution (normal distribution) as shown in Figure 1b and hence increases its correlation coefficient to 0.94 (which is the best among the employed methods). However, with the nth root transformation, an R^2^ value of 0.88 is obtained.

**Figure 2:**
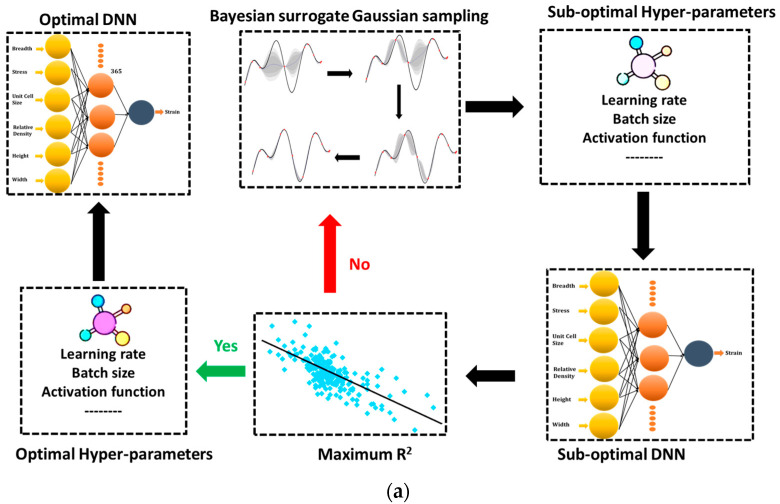

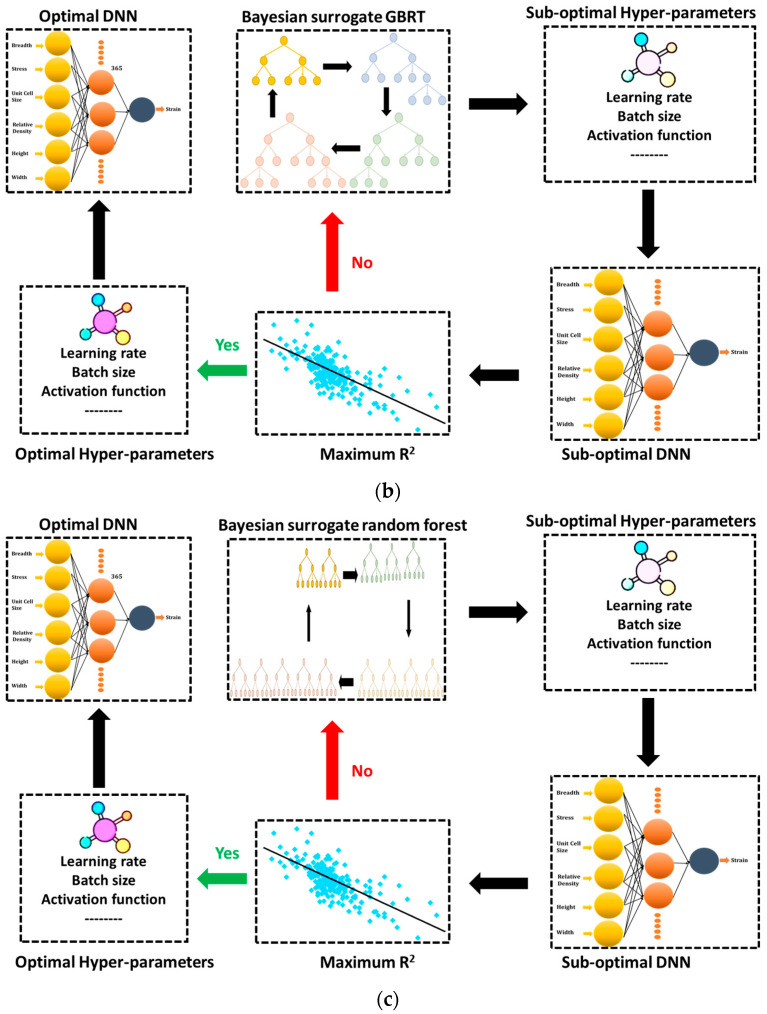
Flow chart of the (**a**) GPR, (**b**) GBRT, and (**c**) RF hyper-parameters optimization schemes.

Gaussian process or GP is an unbounded group of stochastic processes with a constant joint Gaussian distribution in any of the bounded subsets (see Figure 2 for the framework). A mean function and a covariance function are used to represent a GP. The mean function is typically considered to be zero since the GP is a linear combination of random variables with a normal distribution [37,38]. GPR is among the most significant Bayesian ML techniques, and it is built on a very efficient technique for establishing a posterior distribution across a range of features. It is a non-linear regression approach that calculates posterior degradation estimates by limiting the posterior distribution to suit the given training data. It is adaptable enough to tackle issues with high dimensionality, limited sample sizes, nonlinear and complicated logistic regress.

GP is a stochastic process. It is defined as a set of random variables, of which a fixed number have a joint Gaussian distribution. The mean function µ(x) and covariance function C (x, x′) of a GP are entirely defined as,

(1) $$\mu \left(x\right)=E\left[f\left(x\right)\right]$$

(2) $$C\left(x,\;x\prime \right)=E\left[\left(f\left(x\right)-\;\mu \left(x\right)\right)\left(f\left(x\prime \right)-\mu \left(x\prime \right)\right)\right]$$

A GP is defined as, where x, x‘ ∈ X are random variables, and

(3) $$f\left(x\right)\;\sim \;\mathrm{GP}(\left(\mu \left(x\right),\;C\left(x,\;x\prime \right)\right)$$

Gradient boosting, similar to the decision tree, is an aggregation approach that integrates several learning algorithms. A GBRT addressing prediction applications is a mix of gradient boosting and regression trees which employs groups of regression trees to minimize error across a huge single structure, as even the names suggest. The error values created by subtracting the estimated value from the intended target truth value in the first tree are given to the second successive tree. The error values obtained from subtracting the summation of the values obtained in the first and second trees from the primary target truth value are used to build the third tree. This procedure is continued till the parameters reach their maximum value. The final projected value is calculated by adding all the predicted values from decision trees.

The RF algorithm is a collection of binary trees based on two stochastic variables: a randomly generated bootstrap set and a randomly selected set of features for every node. The bootstrap set, which comprises the samples for creating a tree, and the out-of-bag (OOB) set, which contains test examples that are not included in bootstrap set, are used to create trees from the RF. The training instances are randomly sampled from the training set with replacement to create the bootstrap set. Each tree is trained and assessed using its own bootstrap set and OOB set. The splitting criterion used in each node aims to maximize the information gain. The RF employs just a limited number of variables (m_tries_) from all available variables to analyze the information obtained (M). These m_tries_ parameters are picked at random, and only these variables are used to optimize the splitting criterion. The RF optimization technique assesses the so-called OOB error whereas the classifier has been constructed. This error is the average from each tree’s classification error on its individual OOB sets. The OOB error is an unbiased estimate of the classification’s generalized error (GE). The relation of $\mathrm{GE}=\;\rho \left(\frac{1}{S^{2}}-1\right)$ (4) GE was proved by Breiman [39], where s is the ensemble’s strength and ρ is the mean value of correlation. The GE minimum may be achieved by lowering the correlation amongst trees and raising the ensemble classification strength. The aim of RF parameter optimization is determined by these two opposing tendencies.

**Figure 3:**
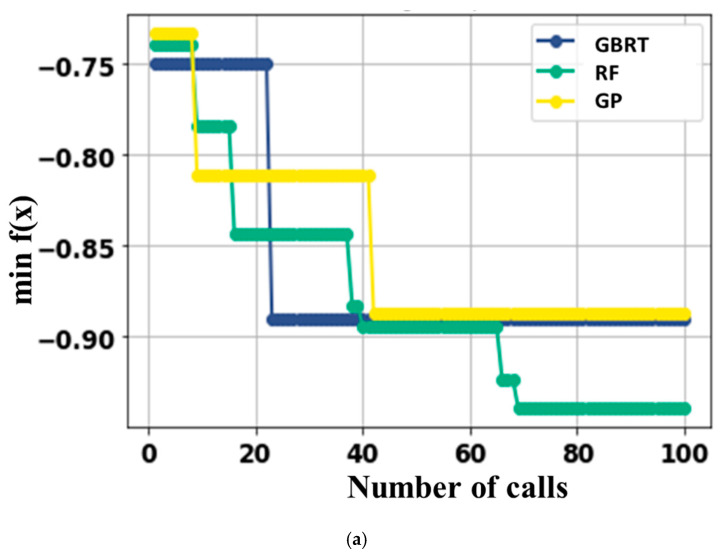

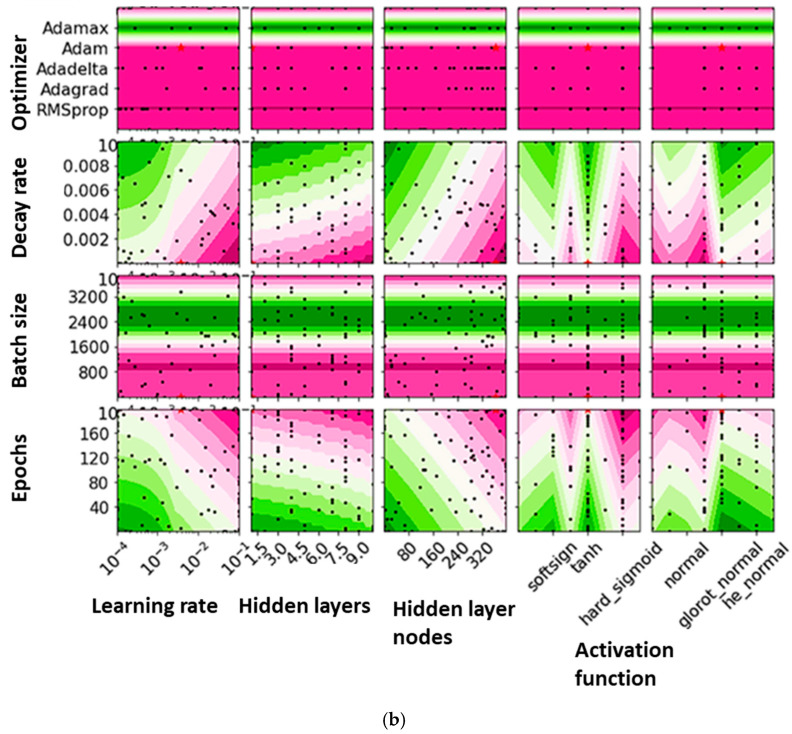
(**a**) Convergence plots and (**b**) hyper-parameters for the Bayesian surrogate models.

**Figure 4:**
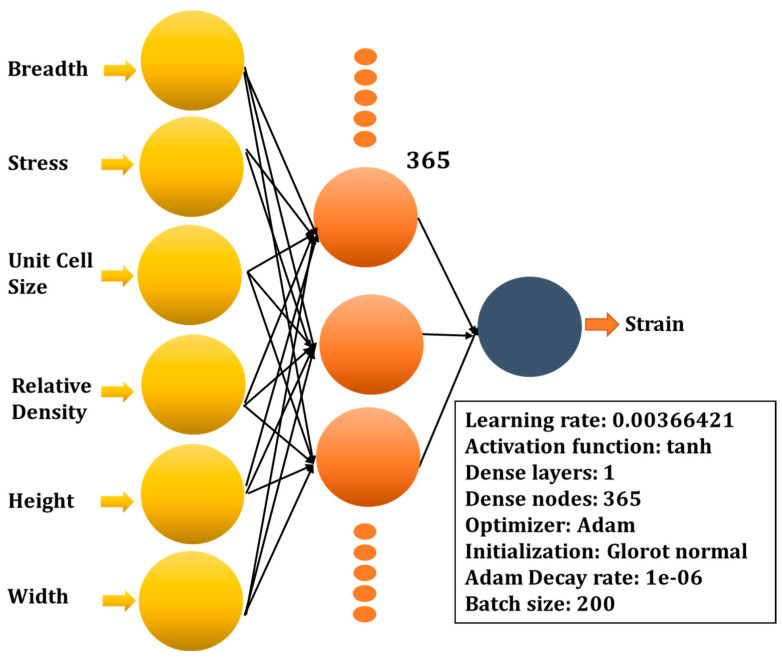
Framework of the optimal DL model.

Convergence plots for the Bayesian surrogate models can be seen in Figure 3a. The convergence of the GBRT, GP, and RF models is shown with respect to the number of calls. Different investigated hyper-parameters (A hyperparameter in machine learning is a parameter that affects how the learning process is carried out.) are provided in Figure 3b and the framework of the optimal deep neural network (DNN) model is represented by Figure 4. The investigated hyper-parameters include learning rate, activation function, number of hidden layers, number of dense nodes in each hidden layer, optimizer, kernel initialization mode, batch size, epochs, etc. Based on the data distribution, transformation, and hyper-parameters tuning, an optimal deep learning model is attained, which has a learning rate of 0.003664218944047891, a single hidden layer containing 365 nodes. The optimal model uses “tanh” as the activation function, “glorot normal” as the initialization mode, “Adam” as the optimizer. The decay rate and batch size for the optimal model are “1e-06” and “200”, respectively.

## 3. Results

Results and discussion sections consists of data correlation (inputs and output), development of deep neural network (DNN), prediction and performance evaluation of DNN model followed by the parameter sensitivity analysis and interpretations of the DNN results by explainable artificial intelligence (XAI).

### 3.1. Correlating the Input and Output Features

**Figure 5:**
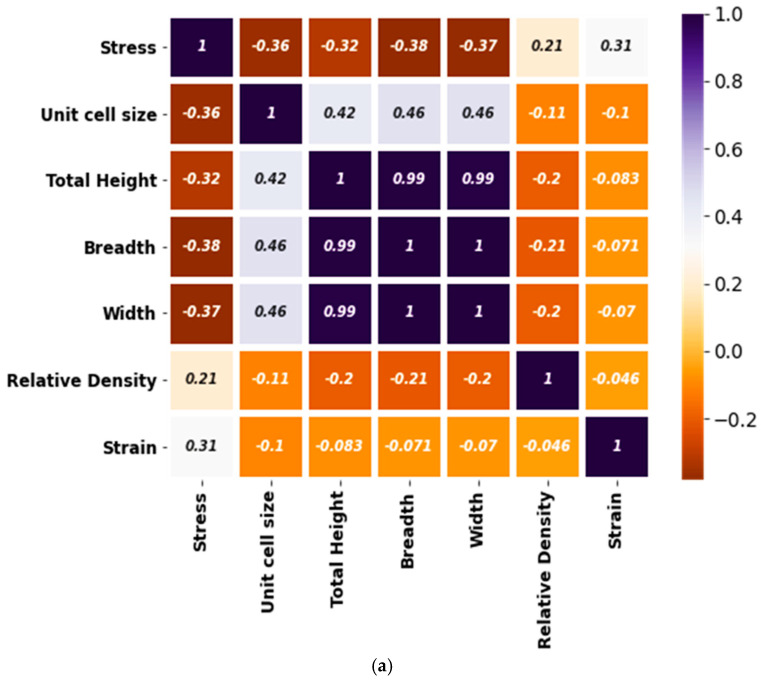

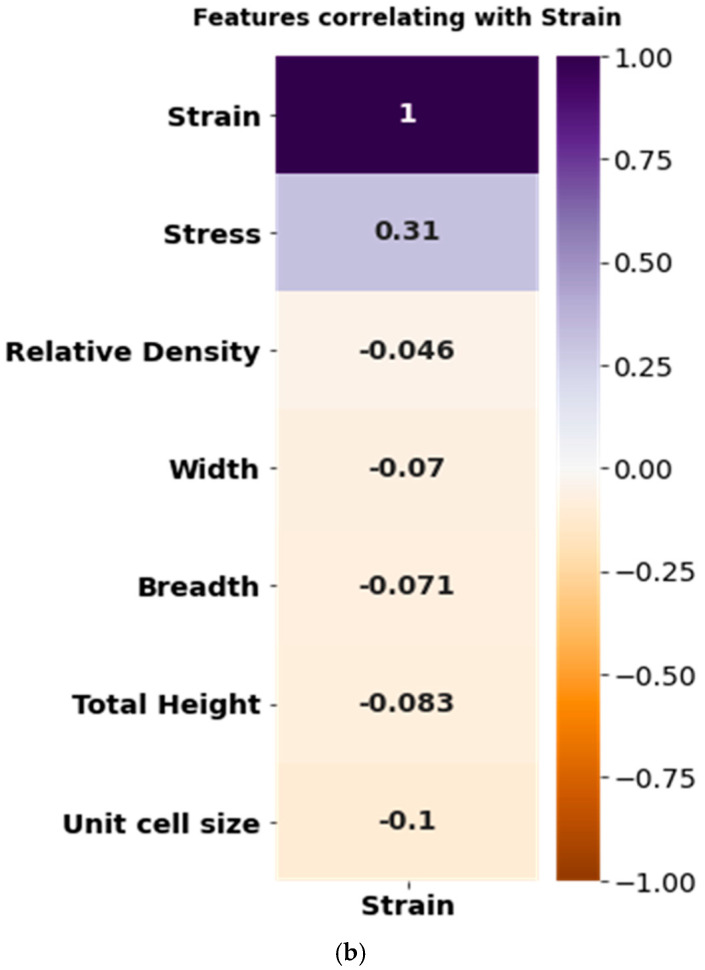
Features correlation with strain (**a**) with inputs and outputs and (**b**) with outputs.

The experimental data of inputs and output is correlated by using Pearson correlation. The input parameters including stress, unit cell size, total height, breadth, relative density, and width are correlated with each other and strain (output parameter) as shown in Figure 5. The details about this are available in a prior study [40]. Figure 5a illustrates the correlation between all of the considered input and out parameters while Figure 5b highlights the correlation of each individual input with the output parameter. As obvious, stress and strain are strongly correlated with each other. In addition, this correlation is positive (or direct). However, similar to the relative density, the dimensions such as height, width, breadth, and unit cell size are relatively weakly correlated with strain (see Figure 5). It can be noted that other than stress, the rest of the input parameters are negatively correlated with the strain for the considered data of additively manufactured lattice structures for a wide range of topologies, materials, and additive manufacturing methods.

### 3.2. Predictive Performance of the Developed Neural Network Model

**Figure 6:**
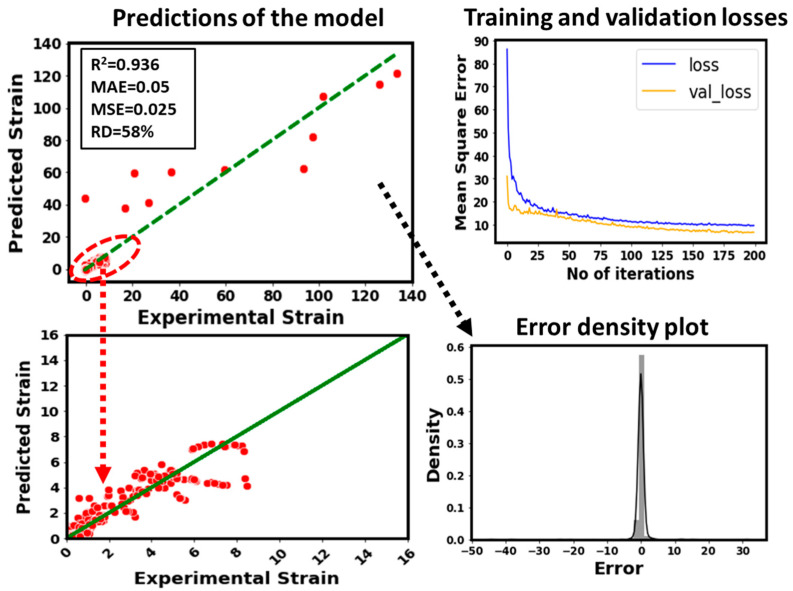
Predictive performance of the developed DL model.

Based on the data correlation and feature engineering, stress, height, width, breadth, unit cell size, and relative density are taken as important and impactful parameters to model the strain of the additively manufactured lattice structures. The optimal framework of the NN is built based on the optimal set of hyper-parameters obtained by GP, GBRT, and RF optimization methods. It can be seen that the developed model predicts the strain with an R^2^ = 0.936, MAE = 0.05, MSE = 0.025, and RD = 58%. Figure 6 clearly depicts that the model’s predictions are more accurate for lower values of strain. This is due to the greater number of data points for this range of the strain. On the contrary, the model’s predictive performance for higher values of strain is poor. This can be attributed to very few data points that have been used for the training. In general, the model’s performance is satisfactory overall. This is because the predictions are made for a wide range of topologies of lattice structures, additive manufacturing methods, materials, and testing ranges.

### 3.3. Parameter Sensitivity Analysis

**Figure 7:**
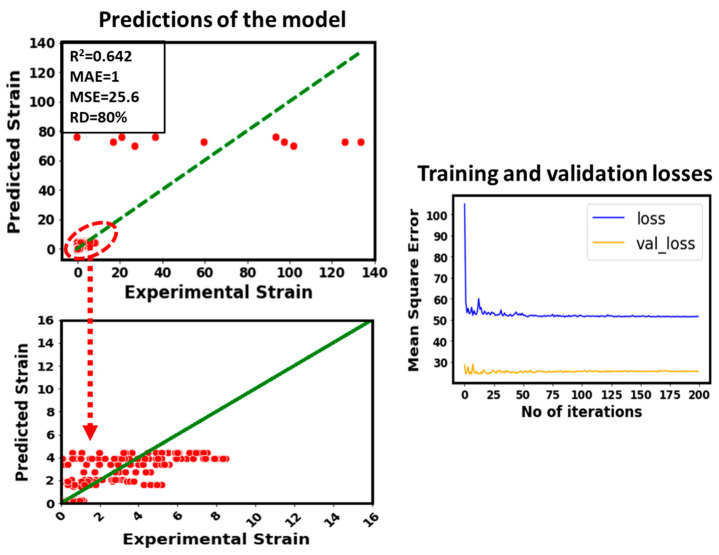
Predictive performance of the developed model by removing stress from the inputs.

In order to find the parametric effect, a sensitivity analysis has been performed based on the model’s predictions. The methodology adopted to perform the deep learning (DL) based parameter sensitivity analysis has been reported in the literature [41]. Herein, the considered input parameters such as stress, lattice dimensions (breadth, width, and total height), relative density, and unit cell size have been dropped from the inputs one by one to visualize their impact on the model’s predictions (e.g., for strain). Figure 7 illustrates the predictive performance of the developed model by removing stress from the inputs. As the number of points between 0 to 16 is very high, so the predictions in this region are zoomed in and shown in another figure. In addition, training and validation losses of the newly developed model (without stress in the inputs) have been shown in Figure 7. Apparently, by removing stress from the inputs, the predictive performance of the developed model becomes very poor and inaccurate.

**Figure 8:**
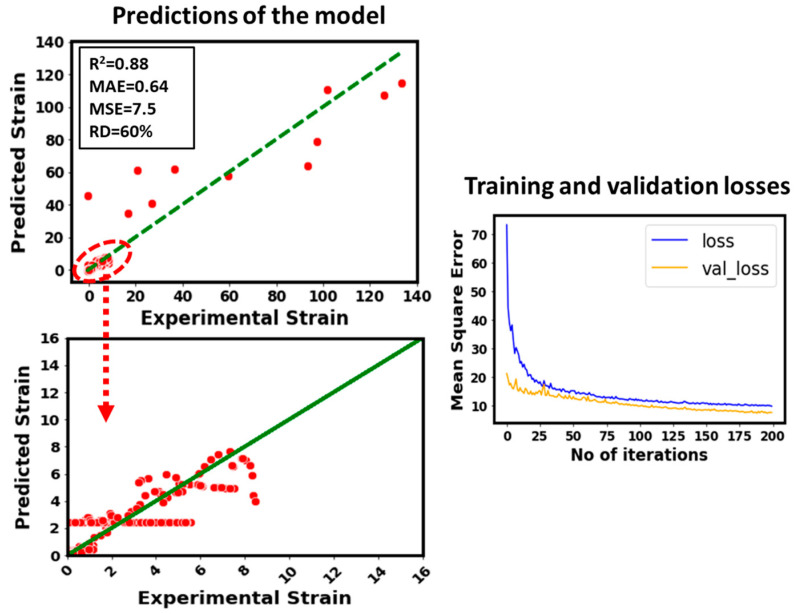
Predictive performance of the developed model by removing unit cell size from the inputs.

For example, the R^2^ value drops from 0.94 to 0.6. This can also be observed from the scattering of the predicted points with respect to the experimental data. The unit cell size is also a very impactful parameter to model the strain of the considered data of additively manufactured lattice structures. This can be witnessed in Figure 8 where unit cell size has been removed from the inputs. It can be seen that the R^2^ value drops from 0.94 to 0.88, and the predicted points are somehow close to the experimental data compared to Figure 7 where stress was removed from the inputs to model the strain. In addition, the training and validation loss plots in Figure 7 and Figure 8 represent this difference.

**Figure 9:**
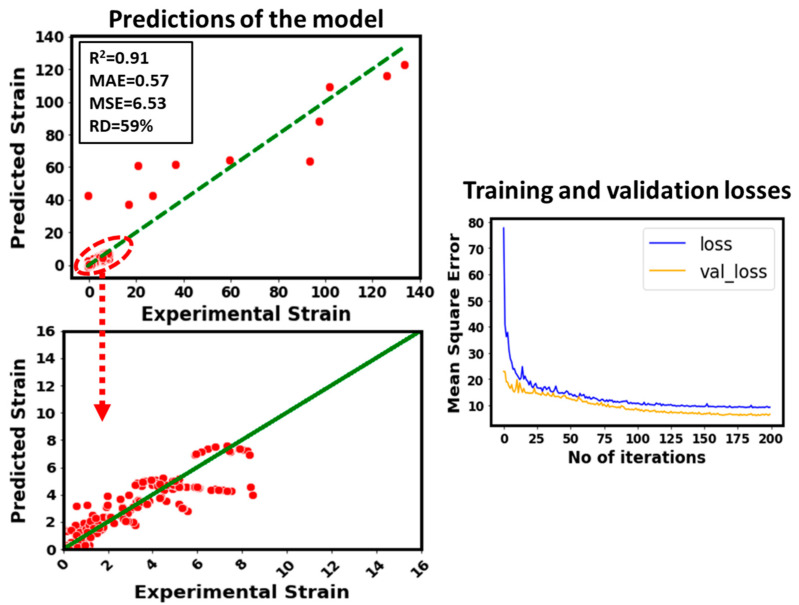
Predictive performance of the developed model by removing lattice dimensions (breadth, width, and total height) from the inputs.

In order to highlight the impact of different lattice dimensions such as breadth, total height, and width, these parameters are removed from the inputs and the strain is modeled as depicted in Figure 9. In this case, R^2^ drops to 0.91 from 0.94. It shows that these lattice dimensions are relatively less sensitive to the strain of the considered data compared to the stress and unit cell size. Also, the training and validation losses plot provides an evidence of this conclusion.

**Figure 10:**
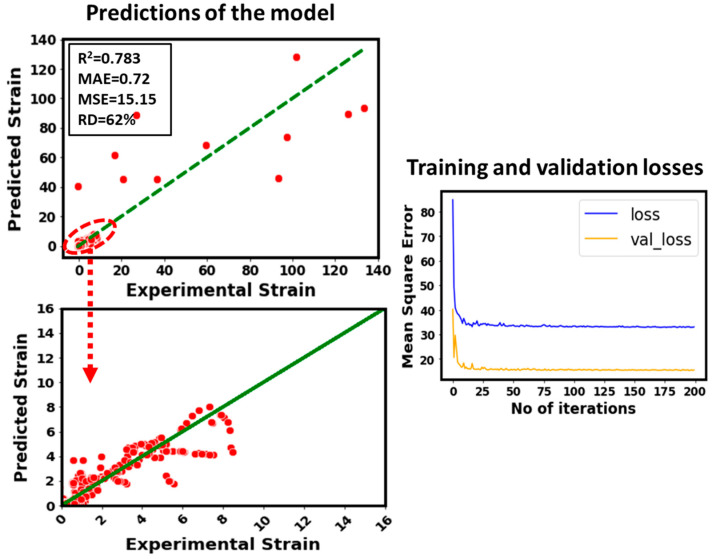
Predictive performance of the developed model by removing relative density from the inputs.

In the end, the impact of relative density on the predicted strain has been quantified (see Figure 10). In this case, relative density has been removed from the inputs list and then the strain has been modeled by using the rest of the input features. It can be noticed that the model’s predicted accuracy becomes poor. For instance, the R^2^ value drops from 0.94 (for original model) to 0.783. This is the second worst model. It means the relative density is the second most sensitive parameter for the strain after the stress. It can be concluded that stress is the most sensitive input to the strain followed by the relative density from the modeling perspective.

### 3.4. Explainable Artificial Intelligence (XAI) Based Insights

**Figure 11:**
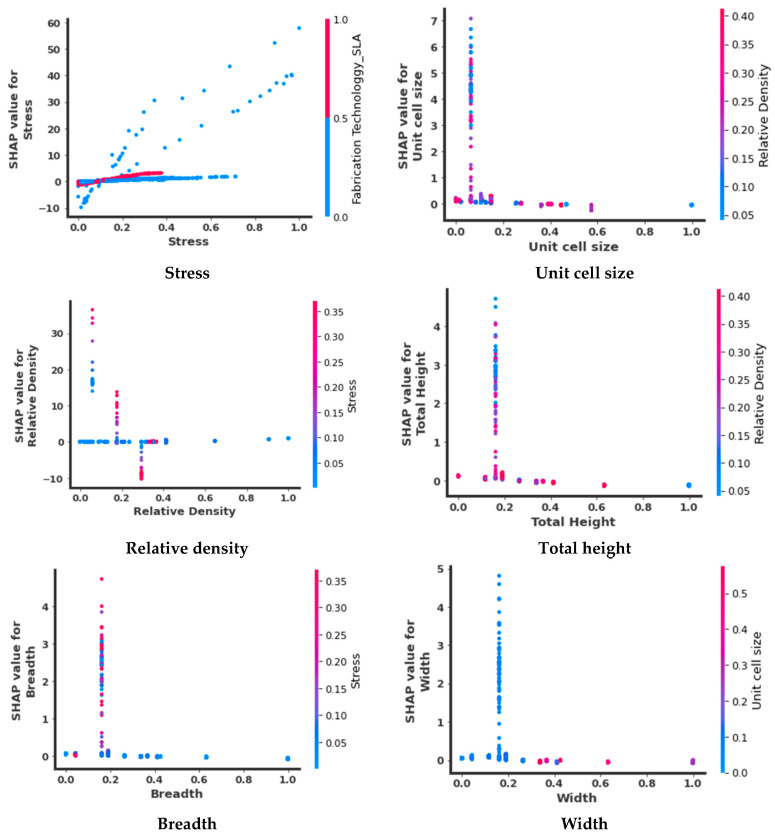
Dependence plots to interpret the predictions of the DL model.

In order to visualize the impact of a single input feature on the prediction ability of the model, a scatter plot known by the name dependence plot can be used. The dots represent the predictions made by the model. The features value is represented by X-axis and Y-axis represents the SHAP value of that feature, which indicates how the prediction changes with the value of the given feature. As seen from Figure 11, as the value of stress increases its shap value increases hence the greater impact on the prediction of the model. The interaction with another feature (chosen automatically by the SHAP dependence plot) is represented by the color of dots. The interaction feature with respect to stress is ‘SLA’ fabrication technology. The shap value of stress with respect to SLA lies at zero (shown in red dots) hence minimum impact on the prediction ability in comparison to other fabrication technologies. The unit cell size, total height, breadth, and width show similar behavior as most impact values lie between 0.0 and 0.2. The rest of the values have lower impact on the prediction of the model. The unit cell size and total height interacts with relative density whereas breadth and width interact with stress and unit cell size respectively. The effective values of relative density lie between 0.0 and 0.4 respectively whereas the rest of the values shows the least impact on the prediction ability of the model. The relative density is interacted with stress. The highest impact of the stress is 0.35 (normalized value between 0 and 1) which lies on the 0.1 value of the relative density.

**Figure 12:**
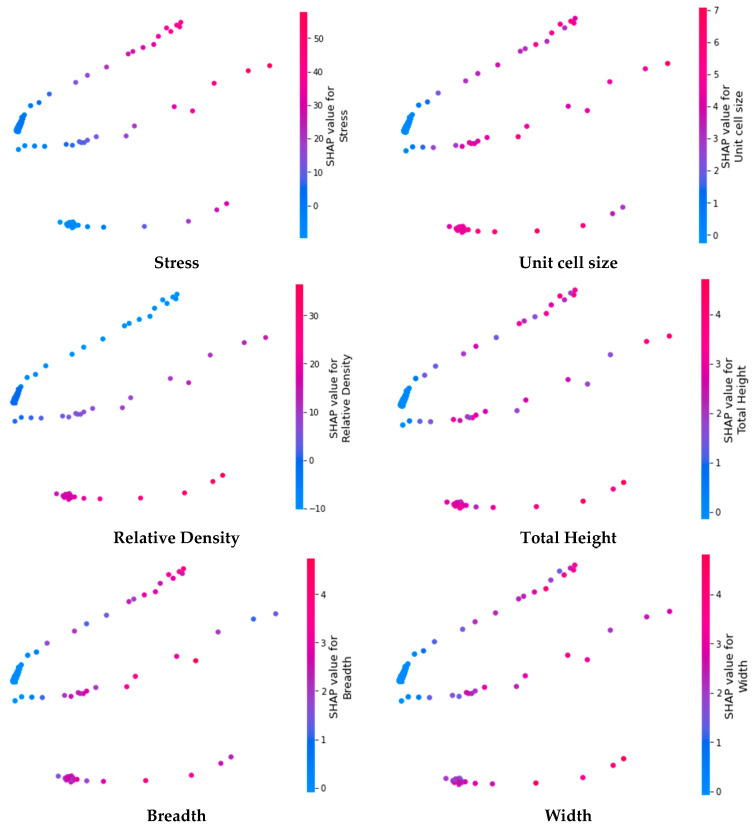
Embedding plots to interpret the predictions of the DL model.

In order to visualize the distribution of SHAP values, an embedding plot is used which is a 2D projection created by PCA (Principal Component Analysis) as shown in Figure 12. It actually visualizes the scattering of SHAP values for a given feature. The embedding plots have been created for all the input features (stress, unit cell size, relative density, total height, breadth, and width) as shown in Figure 12. The stress has the highest impact on the prediction ability as its range lies between −10 to 50. The second most influential parameter is relative density where the distribution of SHAP values lies between −10 to 30. The third most influential parameter is unit cell size where shap values lie between 0 to 7. The other parameters are the least influential as the SHAP values lie from 0 to 4. The importance of features is based on the impact of the feature on the prediction of the model.

**Figure 13:**
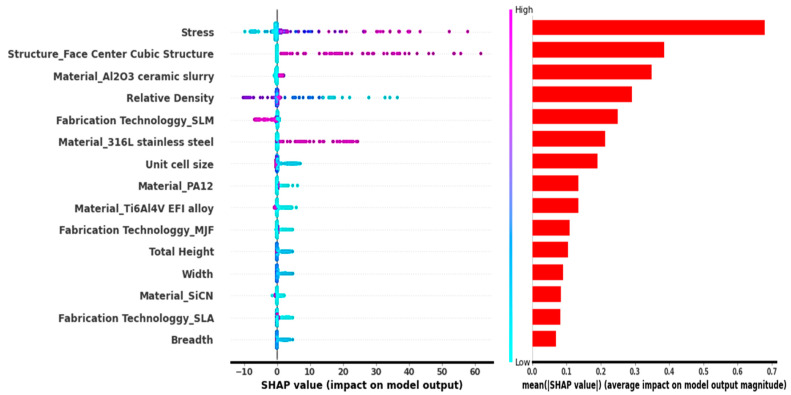
Summary plots to interpret the predictions of the DL model.

The descending order of feature importance is depicted by a summary plot as shown in Figure 13. The x-axis represents the mean value shape and the y-axis represents the given features. Similar to dependence and embedding plots, summary plots also depict that stress is the most influential factor, relative density comes second, unit cell size comes third and dimensions are the least influential parameters. There are categorical features in the plot and this is due to the number of points of the given categorical feature.

**Figure 14:**
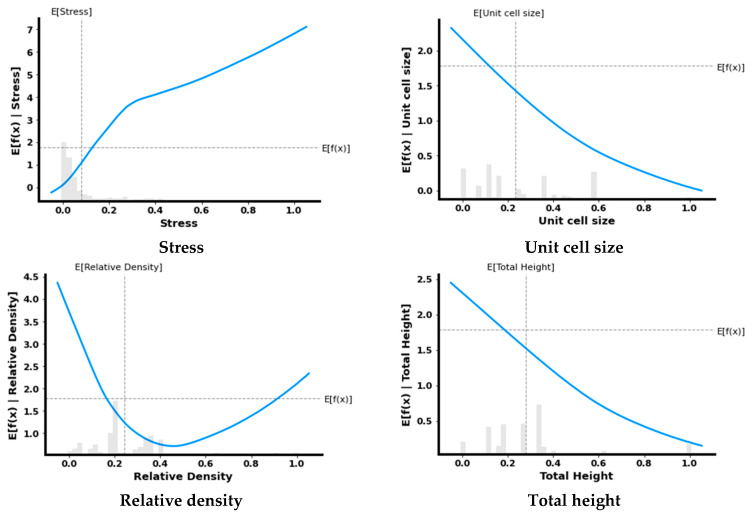

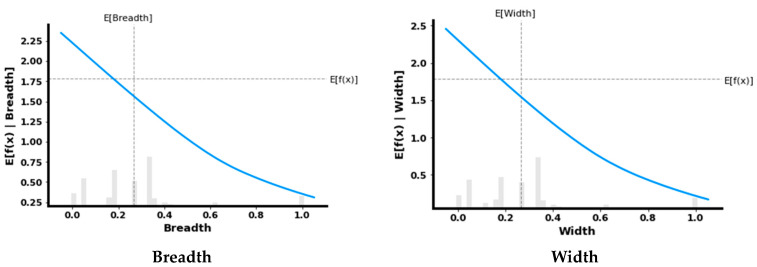
Partial dependence plots to interpret the predictions of the DL model.

Figure 14 depicts the partial dependence plots of several input characteristics. The partial dependence plots demonstrate the influence of a factor while marginalizing all other features. The partial plots illustrate how a parameter should be modified to maximize the influence of a certain characteristic. The horizontal line shows the predicted value, whereas the vertical line reflects the feature’s median. As seen in Figure 14, the most important parameter is stress whose impact increases as its value increases. The impact of relative density first decreases up to 0.4 and then increases up to 1. The impact of other parameters such as unit cell size, total height, breadth and width decreases with respect to its value.

**Figure 15:**
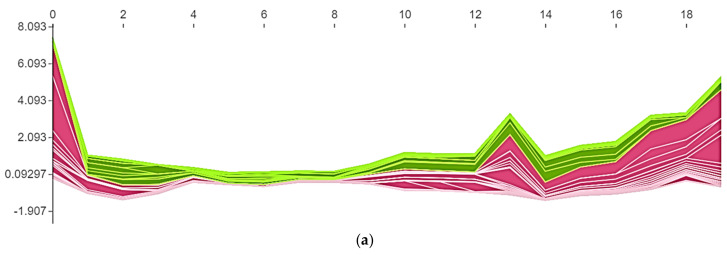

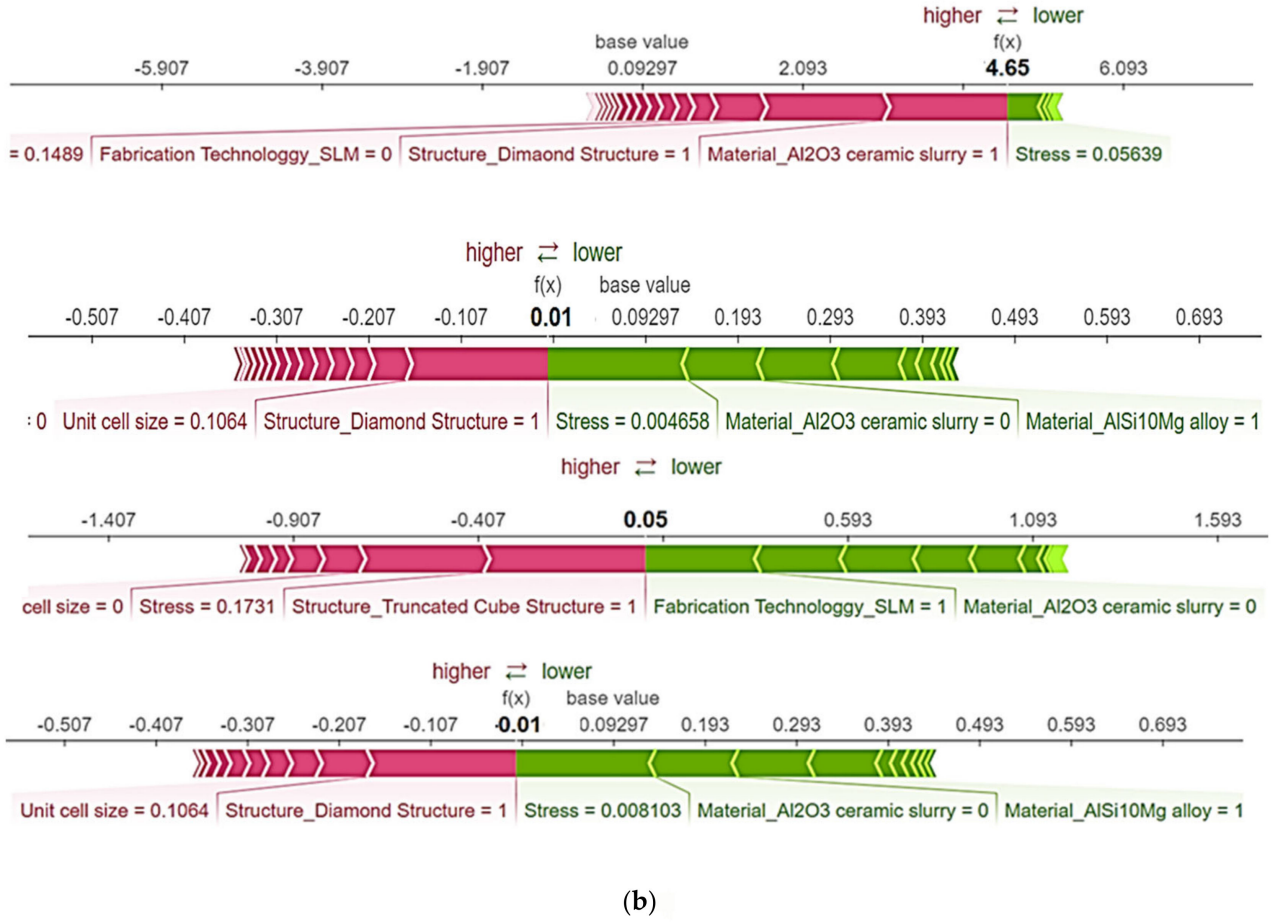
Interpretation of the predictions of the DL model by (**a**) stacked and (**b**) individual force plots.

An additive force layout plot can be generated to visualize prediction by means of SHAP values (see Figure 15). It demonstrates which factors contributed how much favorably or adversely to the basic value in order to make a prediction. Figure 15a is clustered force plot or clustering shap values to visualize the force layout of all the test data. The clustered force plot is shown only for 18 test data for the clear demonstration. Figure 15b–e demonstrates individual force plots for 6 test points. The pink arrows reflect SHAP values that increase the prediction value (to the right), while the green arrows show those that decrease the prediction value (to the left). Each arrow’s size shows the magnitude of the influence of the related characteristic. The “base value” is the model’s average prediction over the test set. Figure 15a is summarized as follows:

1. The predicted value for this observation is 4.65.
2. The base value is 0.09297 (without any effects).
3. The chosen structure is a diamond structure and material is Al_2_O_3_ ceramic slurry.
4. Stress is negatively related to prediction by the amount 0.05639.

Similarly, the other individual force plots can be summarized.

**Figure 16:**
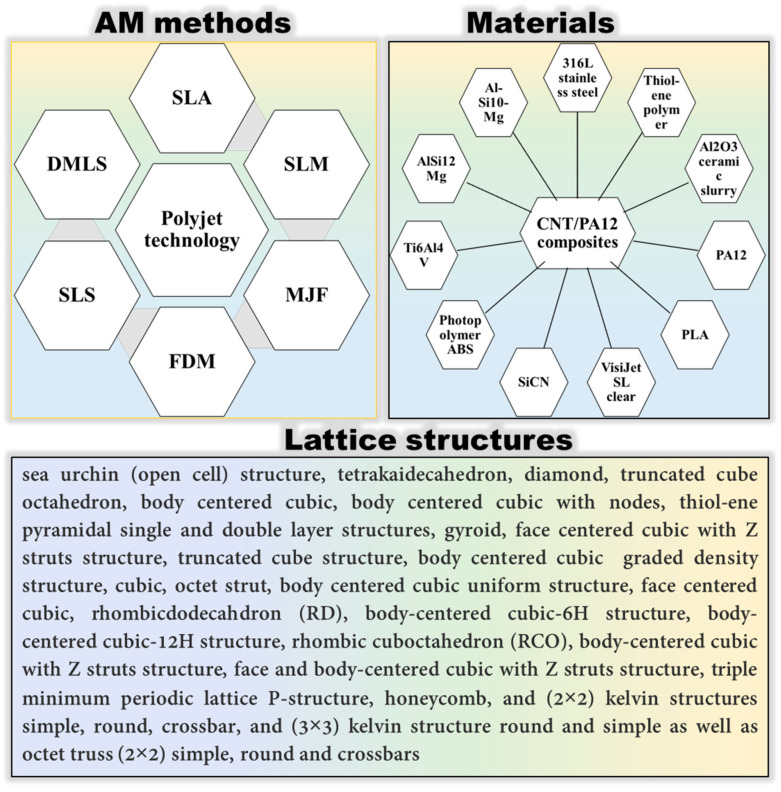
Applicability of the developed method.

From the above discussion and analysis, it can be observed that the employed method is applicable to a wide range of materials, AM methods, and lattice structures (see Figure 16).

## 4. Conclusions

The aim of this work is to evaluate the stress-strain relationship of various AM lattice topologies, to carry out a parameter sensitivity analysis, and to highlight comprehensible AI-based insights for modeling the strain of various AM lattice architectures. As a result, the relationship between a number of AM lattices constructed using various AM techniques, materials, and topologies, and various inputs, including unit cell size, stress, breadth, total height, relative density, and width, has been studied. The major findings are provided as follows.

– The developed model predicts the strain with a R^2^ = 0.936, MAE = 0.05, MSE = 0.025, and RD = 58%.
– Predictability of the developed model is valid for different materials (metals and alloys, composites, ceramics, and polymers) via different AM methods such as FDM, MJF, SLM, SLA, DMLS, polyjet technology, and SLS.
– The developed model can accurately predict the stress-strain relationship in different topologies such as (circular, octagonal, Gyroid, truncated cube, Truncated cuboctahedron, Rhombic dodecahedron, Rhombicuboctahedron, Kelvin lattice structures. 3 × 3 samples, 2 × 2 samples, vertex cube (VC), and edge center cube (ECC), pyramidal truss, periodic cellular, octet-truss, (BCC) body centered cubic, (BCCZ) body centered cubic with Z struts, (FCC) face centered cubic, (FCCZ) face centered cubic with Z struts, (FBCCZ) face and body centered cubic with Z struts, polymeric lattice, triply periodic minimal surface, cubic and diamond structures, CLS struts.
– Parameter sensitivity analysis reveals that stress is the most sensitive input to the strain followed by the relative density from the modeling perspective.
– The explainable artificial; intelligence (XAI) also confirms that the stress has the highest impact on the prediction ability as its range lies between −10 to 50. The second most influential parameter is relative density where distribution of SHAP values lies between −10 to 30. The third most influential parameter is unit cell size where SHAP values lies between 0 to 7. The other parameters are the least influential as the SHAP values lies from 0 to 4. The importance of features is based on the impact of the feature on the prediction of the model.

It is hoped that this methodology will assist researchers and industry in using data-driven techniques to provide preliminary information on the influential parameters prior to conducting trials. Furthermore, this methodology presents a modeling framework for assessing the mechanical properties of the additively created structures and materials under consideration. Although, the proposed scenario covers a wide data range of the additively manufactured lattice structures, yet, this methodology can be extended to additional additive manufacturing methods for other materials and architectures for a wide range of data.

## Figures and Tables

**Table 2:** An overview of the employed methods to find the optimal deep learning model.

S. No.	Description	Max R^2^	Bayesian Surrogate Model
**1**	Simple model	0.85	Forest, Gaussian Process, GBRT
**2**	With Robust Scaler	0.94	Forest, Gaussian Process, GBRT
**3**	With *n*th root transformation	0.88	Forest, Gaussian Process, GBRT

## Data Availability

The data are contained within the article.
